# Unilateral L4-dorsal root ganglion stimulation evokes pain relief in chronic neuropathic postsurgical knee pain and changes of inflammatory markers: part II whole transcriptome profiling

**DOI:** 10.1186/s12967-019-1952-x

**Published:** 2019-06-19

**Authors:** Thomas M. Kinfe, Maria Asif, Krishnan V. Chakravarthy, Timothy R. Deer, Jeffery M. Kramer, Thomas L. Yearwood, Rene Hurlemann, Muhammad Sajid Hussain, Susanne Motameny, Prerana Wagle, Peter Nürnberg, Sascha Gravius, Thomas Randau, Nadine Gravius, Shafqat R. Chaudhry, Sajjad Muhammad

**Affiliations:** 1 Department of Psychiatry, Rheinische Friedrich-Wilhelms University, Sigmund-Freud Street 25, 53105 Bonn, Germany; 2 Division of Medical Psychology (NEMO Neuromodulation of Emotions), Rheinische Friedrich-Wilhelms University, Bonn, Germany; 3 University Hospital Bonn, Rheinische Friedrich-Wilhelms University, Bonn, Germany; 4 Cologne Center for Genomics (CCG), University of Cologne, Cologne, Germany; 5 Institute of Biochemistry I, Medical Faculty, University of Cologne, Cologne, Germany; 6 Center for Molecular Medicine Cologne (CMMC), University of Cologne, Cologne, Germany; 7 Department of Anesthesiology and Pain Medicine, University of California, San Diego, CA USA; 8 San Diego Health Sciences, VA San Diego Healthcare System, San Diego, CA USA; 9 The Spine and Nerve Center of the Virginias, Charleston, WV USA; 10 Volta Research, San Francisco, CA USA; 11 Neuromodulation Specialists LLC, Fairhope, AL USA; 12 Cologne Excellence Cluster on Cellular Stress Responses in Aging-Associated Diseases (CECAD), University of Cologne, Cologne, Germany; 13 Department of Orthopedics and Trauma Surgery, Shifa Tameer-e-Millat University, Islamabad, Pakistan; 14 Dept. of Basic Medical Sciences Shifa College of Pharmaceutical Sciences, Shifa Tameer-e-Millat University, Islamabad, Pakistan; 15 Department of Neurosurgery, University of Helsinki and Helsinki University Hospital, Helsinki, Finland

**Keywords:** Dorsal root ganglion stimulation, Chronic neuropathic pain, Gene expression, Transcriptome

## Abstract

**Background:**

In our recent clinical trial, increased peripheral concentrations of pro-inflammatory molecular mediators were determined in complex regional pain syndrome (CRPS) patients. After 3 months adjunctive unilateral, selective L4 dorsal root ganglion stimulation (L4-DRG_STIM_), significantly decreased serum IL-10 and increased saliva oxytocin levels were assessed along with an improved pain and functional state. The current study extended molecular profiling towards gene expression analysis of genes known to be involved in the gonadotropin releasing hormone receptor and neuroinflammatory (cytokines/chemokines) signaling pathways.

**Methods:**

Blood samples were collected from 12 CRPS patients for whole-transcriptome profiling in order to assay 18,845 inflammation-associated genes from frozen blood at baseline and after 3 months L4-DRG_STIM_ using PANTHER™ pathway enrichment analysis tool.

**Results:**

Pathway enrichment analyses tools (GOrilla™ and PANTHER™) showed predominant involvement of inflammation mediated by chemokines/cytokines and gonadotropin releasing hormone receptor pathways. Further, screening of differentially regulated genes showed changes in innate immune response related genes. Transcriptomic analysis showed that 21 genes (predominantly immunoinflammatory) were significantly changed after L4-DRG_STIM_. Seven genes including *TLR1*, *FFAR2*, *IL1RAP*, *ILRN*, *C5*, *PKB* and *IL18* were down regulated and fourteen genes including *CXCL2, CCL11, IL36G, CRP, SCGB1A1, IL*-*17F, TNFRSF4, PLA2G2A, CREB3L3, ADAMTS12, IL1F10, NOX1, CHIA* and *BDKRB1* were upregulated.

**Conclusions:**

In our sub-group analysis of L4-DRG_STIM_ treated CRPS patients, we found either upregulated or downregulated genes involved in immunoinflammatory circuits relevant for the pathophysiology of CRPS indicating a possible relation. However, large biobank-based approaches are recommended to establish genetic phenotyping as a quantitative outcome measure in CRPS patients.

*Trial registration* The study protocol was registered at the 15.11.2016 on German Register for Clinical Trials (DRKS ID 00011267). https://www.drks.de/drks_web/navigate.do?navigationId=trial.HTML&TRIAL_ID=DRKS00011267

## Background

In line with previously reported observational cohort and randomized-controlled trials we have most recently demonstrated that unilateral selective L4-dorsal root ganglion stimulation (L4-DRG_STIM_) evoked pain relief in complex regional pain syndrome patients (CRPS) and improved functional capacity as well [1–4]. In addition, we observed changes of peripheral circulating inflammatory mediators in different biofluids (saliva and serum). Significantly increased values of pro-inflammatory biomarkers were observed pre- and post L4-DRG_STIM_ for high-mobility group box 1 (HMGB-1), tumor-necrosis factor α (TNF-α) and interleukin (IL)-6 and leptin. Peripheral pro-inflammatory IL-1β was significantly elevated pre-L4 DRG_STIM_, but not post-L4 DRG_STIM_, while anti-inflammatory IL-10 also declined after 3 months of unilateral L4-DRG_STIM_ with oxytocin from saliva increased after 3 months L4-DRG_STIM._ Members of the adipokine superfamily associated with metabolic disorders such as ghrelin and adiponectin remained unchanged [1].

Previous experimental studies in nerve-injury animal models of chronic neuropathic pain have shown neurostimulation therapy reduces hyperalgesia (behavioral testing) coincident with changes in the levels of various neuroinflammatory peptides as a result of altered genetic expression within tissue samples of the spinal cord and dorsal root ganglion [5–8]. So far, the shift of pain-related gene expression and its alteration by spinal modulation has not been assessed in humans treated with DRG-SCS.

Hence, we extended the molecular profiling of our previously published study and attempted to determine gene expression changes in the whole transcriptome. We then attempted to quantify clinical effects of L4-DRG_STIM_ based on genomic considerations as a result of the clinically applied neurostimulation. To the best of our knowledge, this is the first in-human study evaluating gene expression changes of circulating blood cells in CRPS patients treated with selective, unilateral L4-DRG stimulation.

## Methods

The current study represents an extension of the previously published encompassing genetic analysis of whole transcriptome at baseline and after 3 months of L4-DRG stimulation.

An independent internal local ethical research board/committee (IRB-No. 258/15) approved the study. The study was registered at the 15.11.2016 on German Register for Clinical Trials (DRKS ID 00011267).

https://www.drks.de/drks_web/navigate.do?navigationId=trial.HTML&TRIAL_ID=DRKS00011267.

### RNA preparation and whole-transcriptome profiling

During initiation of the sampling processes 1 sample was lost, hence in the current study we have 11 subjects with CRPS who were fully analyzed at baseline and after 3 months of selective L4-DRG stimulation.

RNA was isolated from the frozen blood and whole transcriptome analysis was performed to identify changes in gene expression pre- and post-stimulation. Blood was collected in EDTA tubes and RNA was extracted with the help of NucleoSpin RNA Blood Midi Kit (Cat# 740210.20, Macherey–Nagel GmbH & Co. KG). 100 ng of RNA from each sample were subjected for gene expression analysis using Clariom-S-human array from Affymetrix^®^ (Santa Clara, CA). GeneChip^®^ Hybridization Oven 645 were used for hybridization, GeneChip Fluidics Station 450 for washing and GeneChip Scanner 7G was used for scanning. After scanning, CEL-Files were generated from DAT-Files by Affymetrix^®^ GeneChip^®^ Command Console^®^ software. Data was analyzed by using Affymatrix^®^ Power Tools Programm (version 1.15.1) first subtracting background, followed by normalization, and then summarizing expression. R-Packages Program was used to obtain the differential expression, q-values and quality check.

The raw data was log transformed before the statistical analysis was performed. To compare two groups, we performed a Student’s *t* test. A p value of less than 0.05 was considered a significant difference between two groups. The analysis was performed using Graph Pad Prism.

### Bioinformatics

Pathway enrichment of differentially expressed genes, obtained from gene expression assay, was performed by two different prediction tools: GOrilla^®^ (Gene Ontology enRIchment anaLysis and visuaLizAtion tool) and PANTHER^®^ V. 14.0 (Protein ANalysis THrough Evolutionary Relationships; http://www.pantherdb.org). This software was used for transcriptome profiling allowing rapid analysis and standardized interpretation of large data in a fully automated workflow [9].

The PANTHER database represents a classification of peptide-coding genes (104 sequenced genome) according to the evolutionary relationships of peptide function included in the Gene Ontology (GO) Phylogenetic Annotation Project and Biological Pathways databases. This includes a phylogenetic tree according to the ontological function, which in turns allows to identify ontologically related peptide subfamilies based upon the hidden Markov model (HMM). The PANTHER model also enables the use of distinct analysis tools (gene list enrichment, Reactome^®^ database) to characterize peptide-coding genes based on the molecular function, biological processes and pathways [10–15]. Finally, the most significantly altered genes were screened and a heat map of upregulated (green) and down regulated genes (red) was prepared.

## Results

### Clinical effects of L4-DRG_STIM_ on pain, functional capacity and serum levels of inflammatory markers

As reported in our previous study, after 3 months of selective L4-DRG_STIM_ pain levels significantly declined (mean NRS; pre-DRG_STIM_: 74.90 ± 16.3 versus 1-week DRG_STIM_: 42.50 ± 13.18 versus 3 months DRG_STIM_: 46.65 ± 27.52; p = 0.003) with improved mood and sleep quality. Pro- and anti-inflammatory serum cytokines concentrations were higher in CRPS subjects compared to healthy controls and remained increased post L4-DRG_STIM_ (HMGB-1, TNF-α, IL-6, leptin) with the exception of circulating pro-inflammatory IL-1β. Serum anti-inflammatory IL-10 declined and saliva neuropeptide oxytocin concentrations increased after L4-DRG_STIM_.

Table 1 summarizes baseline characteristics and applied DRG stimulation pattern and Table 2 the peripheral levels of inflammatory mediators assessed in our previous study [1].

**Table 1:** Distribution of patient characteristics at baseline including demographics, pain intensity, functional state, previous spine surgery and electrode type, spine level of SCS implantation, stimulation parameters, and tolerance threshold values

N	Gender Age	Spine level	Activate contacts	Frequency Hz	Pulse width (μs)	Amplitude (μA)	CPSP diagnosis	BMI	BDI bs	BDI as	PSQI global bs	PSQI global as
1	W (75)	L4—left	1−; 3+	20	300	900–1200	CRPS	34,4	18	7	14	12
2	M (71)	L4—right	1−; 2+	20	250	400–500	CRPS	28	10	6	2	4
3	W (65)	L4—left	1+; 3−	20	400	500–600	CRPS	35,2	14	3	4	5
4	W (72)	L4—right	2+; 4−	20	300	500–700	CRPS	24,1	6	5	5	5
5	M (56)	L4—right	3+; 4−	20	200	1400–1600	CRPS	23,4	17	9	9	7
6	W (50)	L4—left	1−; 2+	20	200	1200–1500	CRPS	26,8	21	10	12	5
7	M(58)	L4—left	1−; 3+	20	300	600–750	CRPS (negative trial)	40,4	21	23	16	4
8	M (63)	L4—left	3+; 4−	20	200	400–1000	CRPS	35,8	20	7	11	6
9	W (50)	L4—left	–	–	–	–	Causalgia (implant failure)	25,8	30	33	9	13
10	W (79)	L4—right	3+, 4−	20	250	700–1400	CRPS	24,7	9	8	20	17
11	W (53)	L4—right	2+, 3−	20	200	300–900	CRPS	28,2	13	11	11	12
12	W (71)	L4—left	1−, 2+	20	200	350–550	CRPS	25,2	21	12	18	15

**Table 2:** Baseline and follow-up values of peripheral concentrations (given in pg/ml, exceptional for HMGB-1 ng/ml) of pro- and anti-inflammatory mediators

Pat. no.	HMGB1_bs	HMGB1_as	IL-1β_bs	IL-1β_as	TNFα_bs	TNFα_as	IL-6_bs	IL-6_as	IL-10_bs	IL-10_as
1	1.956	2.368	0.136	0.065	2.414	2.318	2.759	2.083	22.667	0.000
2	5.897	5.574	0.140	0.070	2.136	2.291	3.524	6.800	32.000	0.000
3	1.838	4.309	0.117	0.201	1.886	1.886	2.566	4.455	25.333	0.000
4	4.397	2.691	0.229	0.089	1.259	1.350	3.862	0.897	40.000	16.667
5	38.691	5.338	0.369	0.201	1.914	1.986	12.041	2.855	0.667	14.667
6	4.515	2.897	0.014	0.056	0.986	1.059	2.490	1.793	0.667	0.000
7	6.868	4.338	0.336	0.350	1.877	1.918	15.462	17.614	7.333	24.000
8	9.132	1.544	0.140	0.103	1.545	1.500	4.462	3.359	76.000	10.667
9	2.485	1.662	0.121	0.093	1.605	1.818	2.090	1.352	94.000	1.333
10	9.868	11.838	0.103	0.131	1.936	1.236	12.903	16.193	59.333	4.667
11	3.603	4.544	0.131	0.173	1.423	1.477	2.566	5.690	60.667	6.000
12	2.544	4.397	0.065	0.150	1.691	1.709	2.538	3.407	38.000	13.333

Pat. no.	Ghrelin_bs	Ghrelin_as	Adiponectin_bs	Adiponectin_as	Leptin_bs	Leptin_as	BDNF_bs	BDNF_as	Oxytocin_bs	Oxytocin_as
1	3895.376	5059.584	10,670	11,340	258,300	219,500	45,790.48	53,552.38	39.7	59.9
2	2622.864	2387.712	8610	8200	11,100	17,000	44,266.67	44,933.33	33.7	42.5
3	4873.856	3762.032	4440	4190	70,600	75,200	34,838.10	23,266.67	22.5	49.5
4	4134.976	13045.936	8730	19,330	49,800	28,200	29,457.14	41,409.52	31.3	15
5	4203.376	3793.04	2110	4300	5600	7400	54,838.10	47,361.90	15	111.2
6	6972.928	8809.568	8760	12,500	23,800	7700	23,076.19	33,980.95	23.9	87.6
7	2374.272	2223.344	6860	3460	40,500	85,000	34,266.67	36,504.76	20.7	15
8	2858.688	2493.792	1650	1300	63,000	56,100	27,742.86	29,933.33	23.1	116.1
9	14,335.92	12126.912	10,620	7230	54,300	45,800	41,171.43	39,266.67	28.7	15
10	10885.504	5767.136	7800	6270	45,300	47,900	35,314.29	42,076.19	15	17.5
11	4144.784	4280.784	28,850	22,450	31,800	36,000	49,742.86	34,647.62	61.4	24.6
12	2387.712	1824.88	4250	3610	135,000	105,900	52,600.00	37,457.14	39.5	119.1

Gravius et al. [1]

bs, before stimulation; as, after 3 months L4-DRG_STIM_

### Transcriptome analyses

**Fig. 1:**
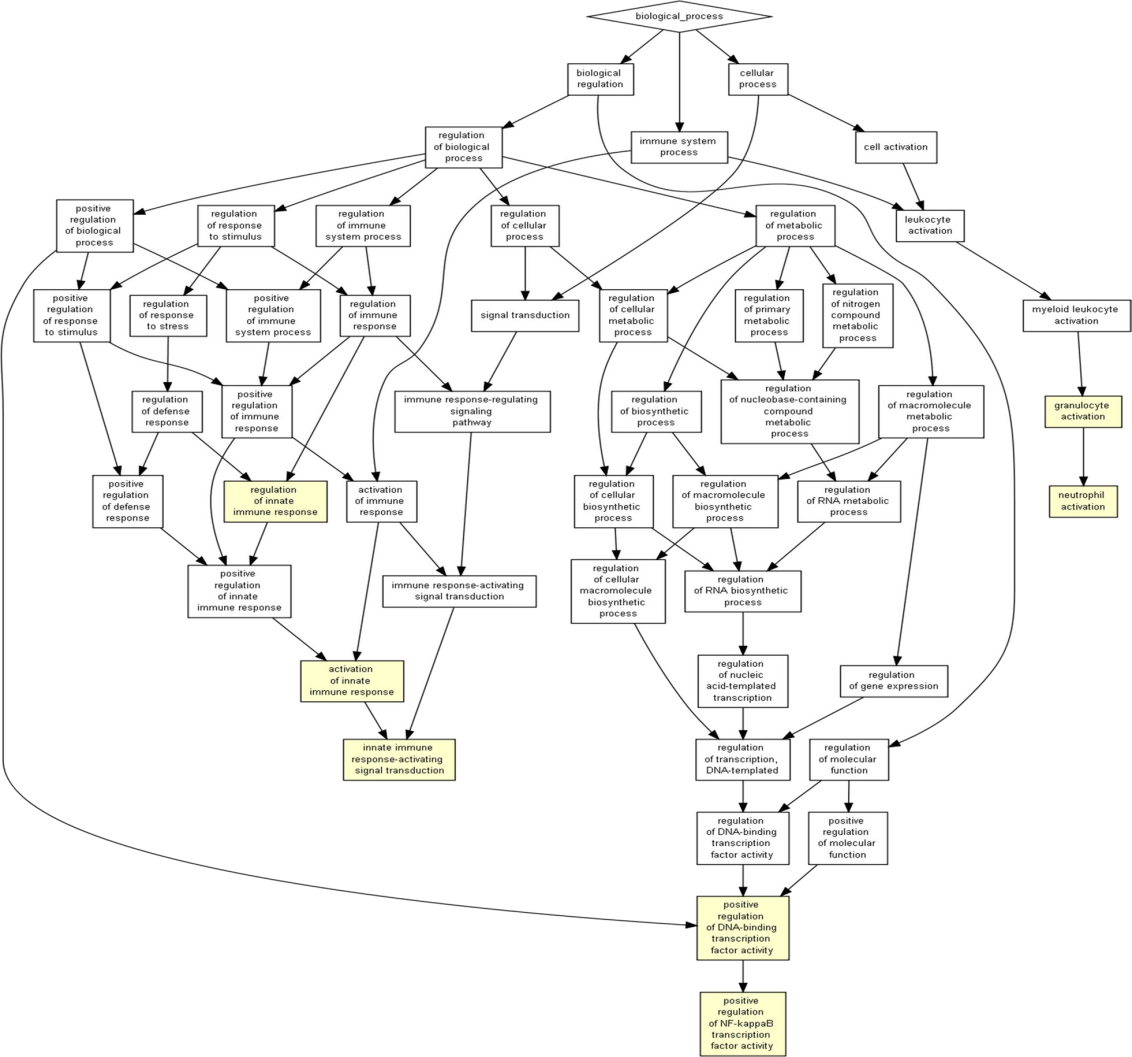
Pathway enrichment for biological processes showing predominantly changes in genes involved in immunoinflammatory pathways after L4-DRG stimulation

**Fig. 2:**
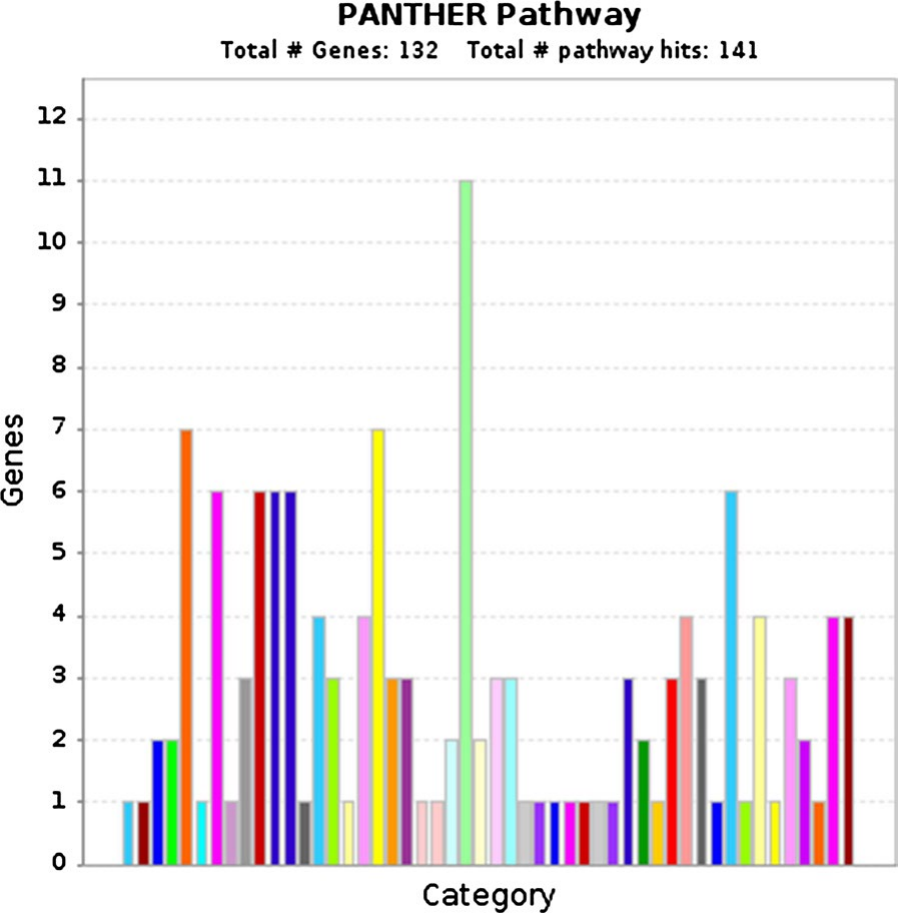
Pathway enrichment of (PANTHER pathway) for 132 screened genes showed predominant involvement of inflammation mediated by chemokine and cytokine signaling pathway (Yellow: Gonadotropin releasing hormone receptor pathway, Green: Inflammation mediated by chemokine and cytokine signaling pathway)

Pathway enrichment analyses showed predominant involvement of inflammation mediated chemokines/cytokines pathway and gonadotropin releasing hormone receptor pathway (Figs. 1, 2).

**Fig. 3:**
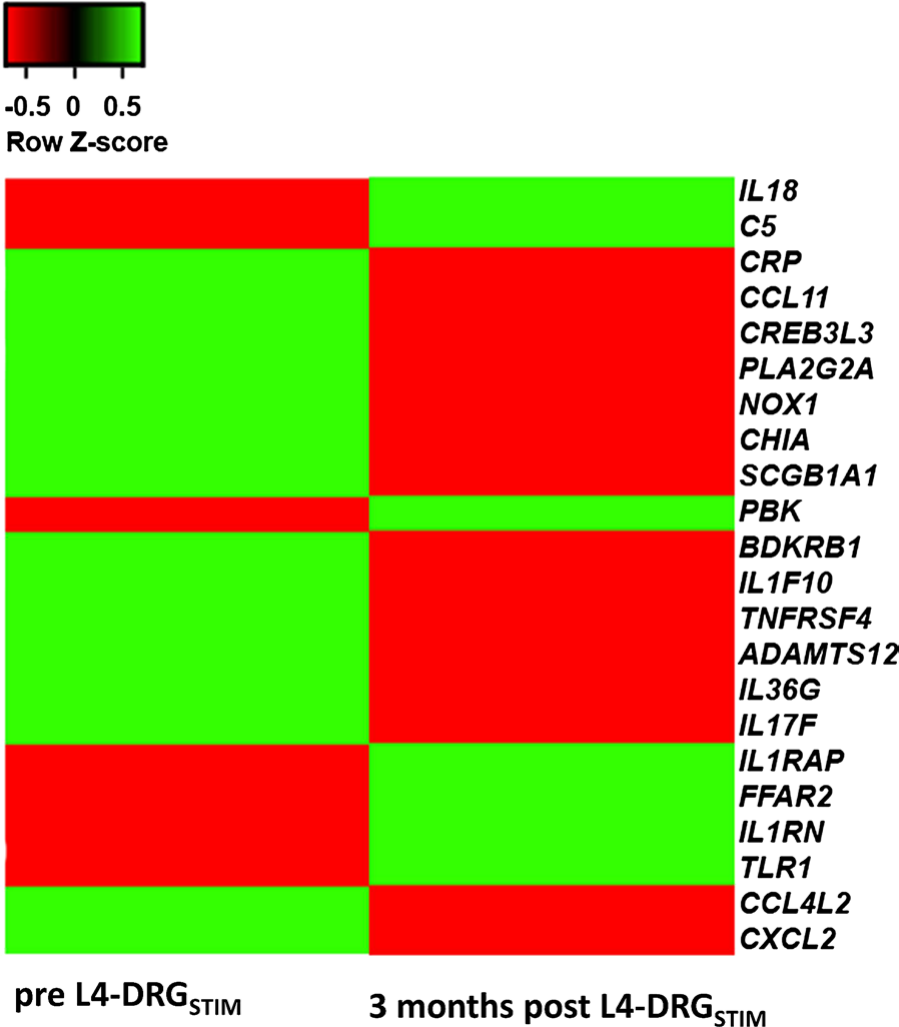
Heatmap showing significantly up-regulated (green) genes for *CXCL2*–*CCL11*–*IL36G*–*CRP*–*SCGB1A1*–*IL*-*17F*–*TNFRSF4*–*PLA2G2A*–*CREB3L3*–*ADAMTS12*–*IL1F10*–*NOX1*–*CHIA*–*BDKRB1* and downregulation (red) genes for *TLR1*–*FFAR2*–*IL1RAP*–*IL1RN*–*C5*–*PKB*–*IL18* after L4-DRG stimulation. p value < 0.05 was considered significant

**Fig. 4:**
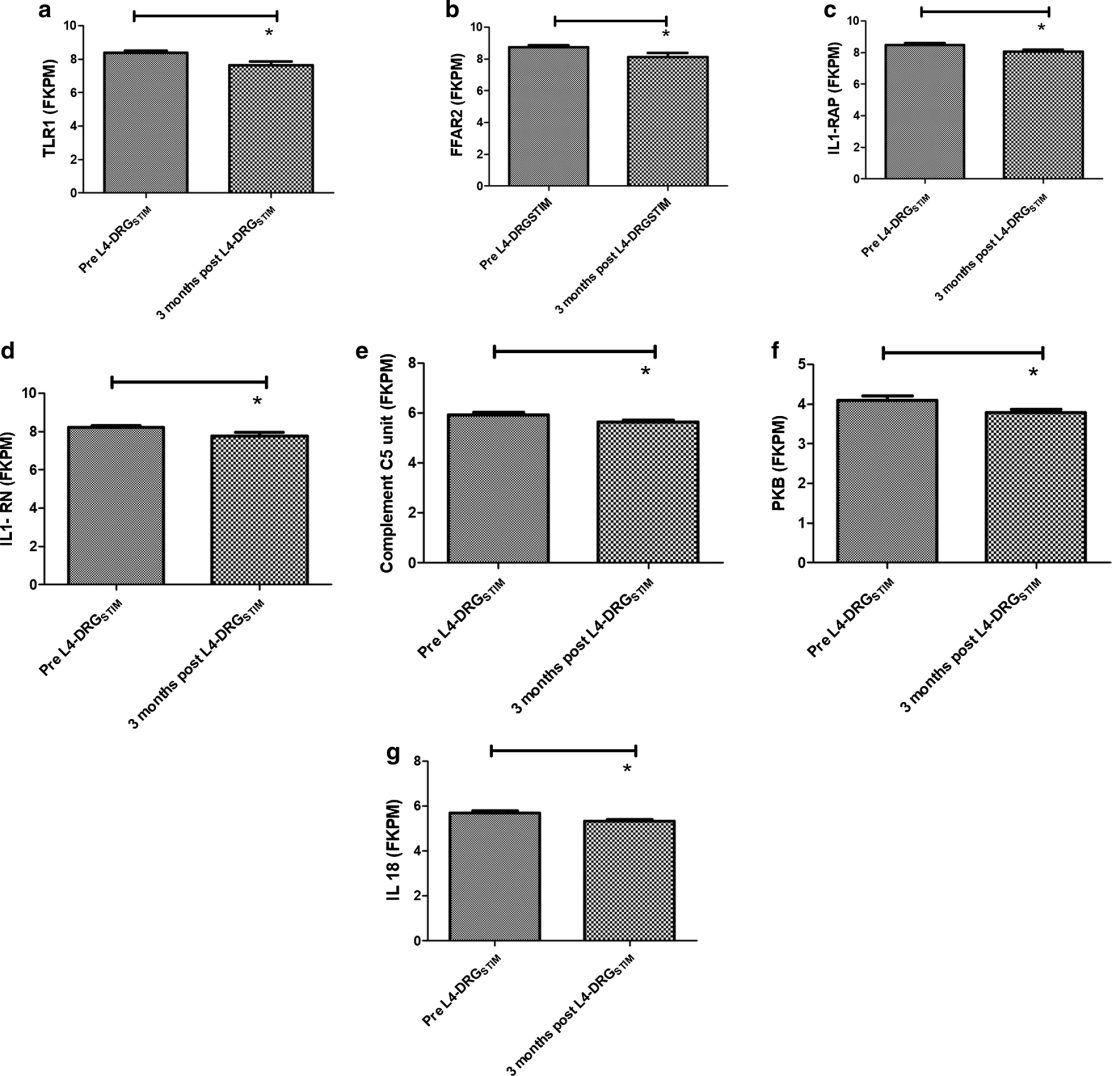
Graphical presentation of genes that were significantly down-regulated *TLR1*–*FFAR2*–*IL1RAP*–*IL1RN*–*C5*–*PKB*–*IL18* after L4-DRG stimulation. p value < 0.05 was considered significant. Asterisk indicate the statistical significance

**Fig. 5:**
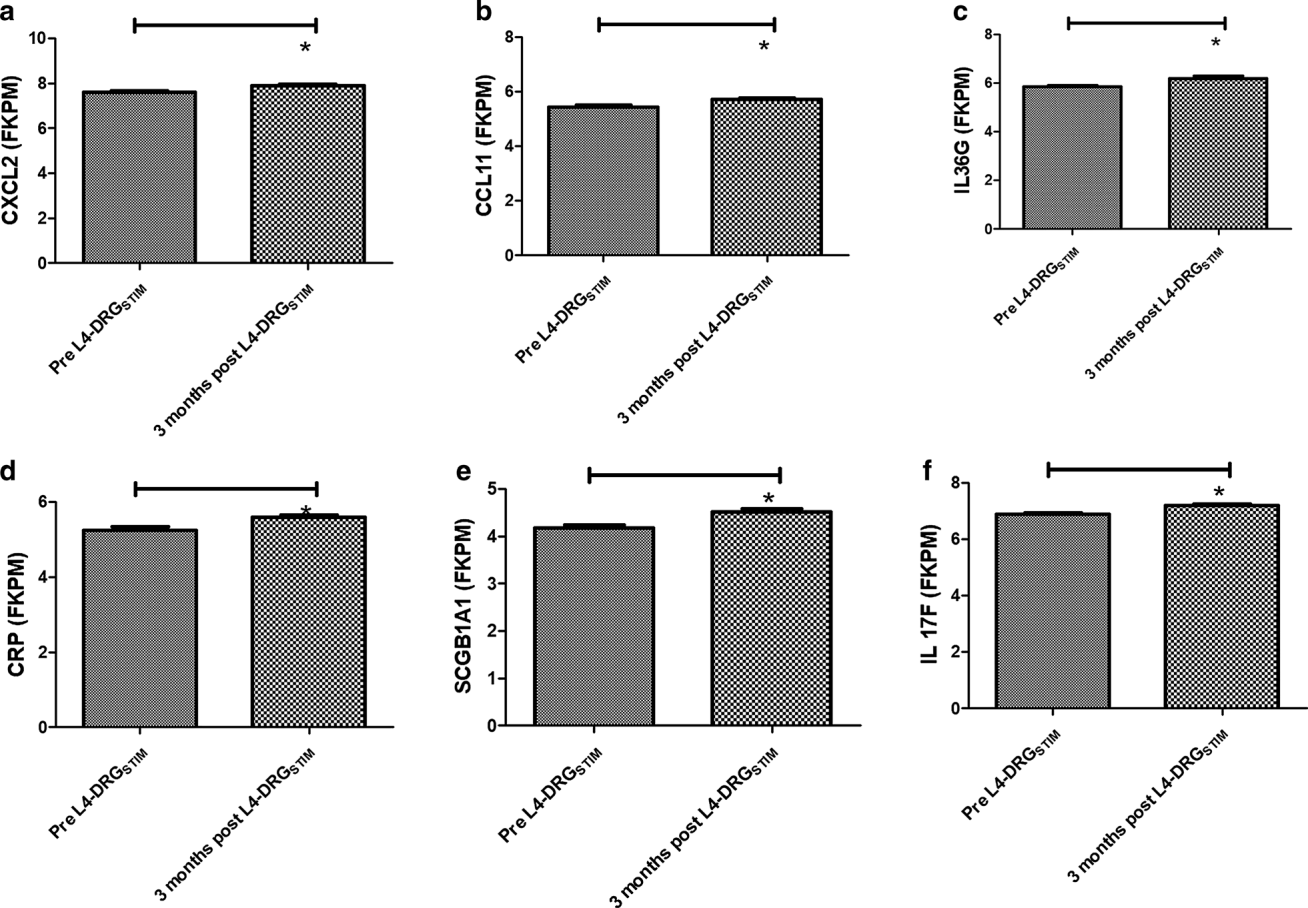
Graphical presentation of genes that were significantly up-regulated *CXCL2*–*CCL11*–*IL36G*–*CRP*–*SCGB1A1* and *IL*-*17F* after L4-DRG stimulation. p value < 0.05 was considered significant. Asterisk indicate the statistical significance

**Fig. 6:**
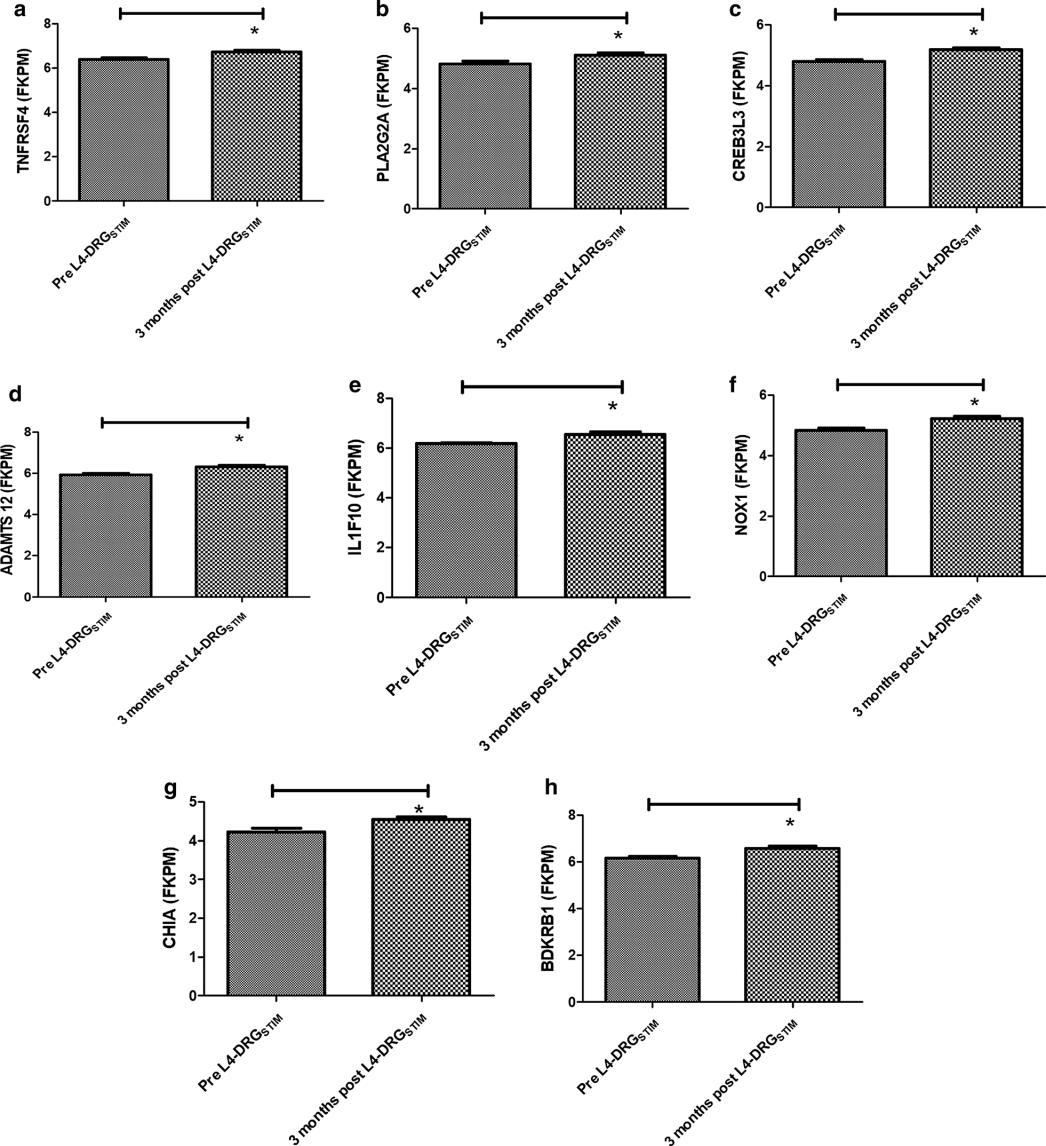
Graphical presentation of genes that were significantly up-regulated *TNFRSF4*–*PLA2G2A*–*CREB3L3*–*ADAMTS12*–*IL1F10*–*NOX1*–*CHIA* and *BDKRB1* after L4-DRG stimulation. p value < 0.05 was considered significant. Asterisk indicate the statistical significance

Further screening of differentially regulated genes showed changes in immune response related genes and genes involved in signal transduction. We observed that after 3 months of L4-DRG_STIM_, 21 genes with predominant involvement of immunoinflammatory responses showed significant differential expression (Fig. 3). Out of these 21 genes, seven [7] genes including toll-like receptor 1 (*TLR1*; − 1.6-fold, p = 0.01), free fatty acid receptor 2 (*FFAR2*; − 1.5-fold, p = 0.04), interleukin 1 accessory protein (*IL1RAP*; − 1.4-fold, p = 0.02), interleukin 1 receptor antagonist (*ILRN*; − 1.3-fold, p = 0.04), complement component 5 (C5; − 1.3-fold, p = 0.01), *PKB* (PDZ binding kinase; − 1.2-fold, p = 0.04) and interleukin 18 (*IL18*; − 1.2-fold, p = 0.05) were downregulated (Figs. 3, 4). The remaining fourteen [14] genes including chemokine (C-X–C motif) ligand 2 (*CXCL2*; 1.2-fold, p = 0.001), chemokine (C–C motif) ligand 11 (*CCL11*; 1.2-fold, p = 0.01), interleukin 36 gamma (*IL36G*; 1.2-fold, p = 0.02), C-reactive protein (*CRP*; 1.2-fold, p = 0.02), secretoglobin, family 1A, member 1 (*SCGB1A1*; 1.2-fold, p = 0.03), interleukin 17F (*IL*-*17F*; 1.2-fold, p = 0.002), tumor necrosis factor receptor superfamily, member 4 (*TNFRSF4*; 1.3-fold, p = 0.006), phospholipase A2, group IIA (*PLA2G2A*; platelets, synovial fluid; 1.2-fold, p = 0.02), cAMP responsive element binding protein 3-like 3 (*CREB3L3*; 1.3-fold, p = 0.006), ADAM metallopeptidase with thrombospondin type 1 motif 12 (*ADAMTS12*; 1.3-fold, p = 0.001), interleukin 1 family, member 10 (theta) (*IL1F10*; 1.3-fold, p = 0.001), NADPH oxidase 1 (*NOX1*; 1.3-fold, p = 0.006), acidic chitinase (*CHIA*) and bradykinin receptor B1 (*BDKRB1*; 1.3-fold, p = 0.003) were upregulated (Figs. 3, 5, 6). The most prominently downregulated gene was *TLR1,* with a − 1.6-fold downregulation (p = 0.01) in comparison to pre-L4 DRG_STIM_. *TLR1* is known to be involved in mediating the inflammatory response through toll-like receptors signaling.

On the other hand, the most prominent upregulated (1.3-fold; p = 0.003) gene was *BDKRB1* that is involved in leukocyte migration and immunoinflammatory response through G-protein coupled receptor signaling. The details of intensity of up or downregulation of each gene have been summarized in Table 3.

**Table 3:** Baseline and post L4-DRG stimulation changes (given in fold) of gene expression demonstrating significant upregulation (green) for *CXCL2*–*CCL11*–*IL36G*–*CRP*–*SCGB1A1*–*IL*-*17F*–*TNFRSF4*–*PLA2G2A*–*CREB3L3*–*ADAMTS12*–*IL1F10*–*NOX1*–*CHIA*–*BDKRB1* and downregulation (red) *TLR1*–*FFAR2*–*IL1RAP*–*IL1RN*–*C5*–*PKB*–*IL18*

Gene	Baseline (FKPM)	SD	L4-DRG stimulation (FKPM)	SD	Fold change	p value
*TLR1*	8.38	0.739	7.73	0.42	− 1.57	*0.011*
*FFAR2*	8.75	0.817	8.2	0.40	− 1.46	*0.038*
*IL1RAP*	8.5	0.506	8.07	0.31	− 1.35	*0.023*
*IL1RN*	8.22	0.566	7.83	0.28	− 1.31	*0.035*
*C5*	5.94	0.252	5.6	0.29	− 1.26	*0.026*
*PBK*	4.1	0.257	3.79	0.37	− 1.23	*0.037*
*IL18*	5.69	0.266	5.39	0.39	− 1.23	*0.021*
*SMAD1*	5.91	0.237	5.63	0.38	− 1.22	0.068
*NAIP*	10	0.360	9.74	0.36	− 1.2	0.089
*CXCL2*	7.61	0.186	7.86	0.14	1.2	*0.0007*
*CCL11*	5.43	0.199	5.69	0.25	1.2	*0.012*
*IL36G*	5.84	0.263	6.12	0.22	1.21	*0.0028*
*CRP*	5.25	0.223	5.52	0.28	1.21	*0.0058*
*SCGB1A1*	4.17	0.233	4.45	0.25	1.21	*0.0042*
*FASLG*	6.52	0.276	6.8	0.32	1.21	0.08
*IL17F*	6.89	0.185	7.19	0.14	1.23	*0.0004*
*TNFRSF4*	6.4	0.218	6.7	0.24	1.23	*0.0033*
*PLA2G2A*	4.82	0.243	5.13	0.30	1.24	*0.026*
*CREB3L3*	4.79	0.206	5.11	0.24	1.25	*0.0006*
*ADAMTS12*	5.94	0.203	6.29	0.20	1.27	*0.0003*
*IL1F10*	6.19	0.284	6.55	0.10	1.28	*0.0009*
*NOX1*	4.83	0.245	5.19	0.29	1.28	*0.0037*
*CHIA*	4.22	0.174	4.58	0.35	1.28	*0.012*
*BDKRB1*	6.16	0.323	6.54	0.22	1.3	*0.0022*
*CCL4L2*	7.49	0.370	7.88	0.41	1.31	0.055
*PTGDR*	8.46	0.306	8.86	0.45	1.32	0.052

p value < 0.05 was considered significant

## Discussion

### Brief summary of our study findings and description of the biological function of the transcripts

Orlova and colleagues examined small-noncoding RNAs (miRNAs; micro-RNAs) in blood and compared CRPS subjects versus healthy controls. Significantly increased levels were observed for ILRa, vascular endothelial growth factor and monocyte chemotactic protein (MCP-1). In addition, miRNAs expression correlated with co-morbidities frequently observed in CRPS such as headache, thyroid disorders and/or use of narcotics/antiepileptic drugs indicating biomarkers having a potential for patient stratification in CRPS associated pain [16]. However, sufficiently powered clinical studies relating the effects of DRG_STIM_ in CRPS patients assessing potentially therapeutic changes in gene expression (e.g. alterations in cytokine and chemokines levels and ratios) have been lacking.

SCS impacts pain signaling in CRPS via inhibiting GABA-ergic and cholinergic interneurons of the spinal cord organized in functional compartments (Laminae I–V). The pathological vascular state in CRPS has been associated by orthodromic (inhibition of the sympathetic tone) and antidromic (activation of spinal afferents) spinal routes [17, 18]. In a sophisticated hypothesis Russo and colleagues discussed possible relevant pathways addressed to the development of CRPS. Briefly summarized, the authors speculated, that in a time-dependent manner CRPS pathophysiology may be the consequence of a deteriorated complex interplay initially driven by local cellular and molecular players (T-cells, B-cells, dendritic cells, cytokines), ultimately leading to microglia activation in the CNS, which in turns promotes peripheral inflammation and drives the maintenance of CRPS. Dendritic cells are known to interact with some of the transcriptional factors of our exploratory study and represent a key cell population with multifunctional attributes including local inflammation synthesis and binding, migration via lymphatic nodes, systemic blood circulation, blood brain barrier penetration via meningeal, choroid plexus and periventricular space related pathways in the CNS. Based upon this, the described transcriptional alterations in our trial appear to have potential validity [18].

Our analysis of CRPS subjects revealed 21 immunoinflammatory-related genes were differentially expressed and significantly changed after L4-DRG_STIM_. Seven (7) genes were significantly downregulated (Table 3): *TLR1* (integral plasma membrane component; TLR4 signaling), *FFAR2* (G-protein coupled receptor activity; cytokine activity; glucose hemostasis), *IL1RAP* (innate immune response; IL-1 receptor activity regulation), *ILRN* (inhibition of glutamate secretion; metabolic processing; response to starvation; insulin secretion; sensory perception of pain; chronic inflammatory signaling of IL-1), *C5* (inflammatory response; cell chemotaxis; response to stress; leukocyte migration), *PKB* (negative regulation of immune response) and *IL18* (regulation of gene expression; T-helper 1 type immune response; positive regulation of inflammation). Interestingly, interleukin 1 receptor related genes (*IL1RAP* and *ILRN*) associated with IL-1β pre-/post synaptic signaling were found to be downregulated. Of note, in line with previous preclinical and human studies we found significantly increased serum levels of pro-inflammatory IL-1β at baseline, but not after 3 months of L4-DRG_STIM_ compared to healthy controls in our previous trial [1]. TLR is richly expressed on dendritic cells surface processing noxious stimuli derived from bacteria/viruses (PAMP, pathogen associated molecular patterns) or with injury-related molecules. Noxious stimuli activating TLR leads to upregulation of pro-inflammatory mediators evoking a host response from the innate and adaptive immune system. The transcriptional downregulation in our cohort may reflect the observed L4-DRG_STIM_ responsiveness. Relational conclusions between such changes and clinical responsiveness are limited in view of the uncontrolled study design, but could be answered in future prospective comparative studies comparing different spinal modulation wave forms.

On the other hand, fourteen (14) genes were upregulated (Table 3). These included *CXCL2* (regulation of cell proliferation extracellular; cell chemotaxis in inflammatory response), *CCL11* (signal transduction; cellular response to IL-1; chemotaxis of neutrophil, mast cells, monocyte and lymphocyte), *IL36G* (positive regulation of IL-6; regulation cytokine synthesis; cell–cell signaling, innate immune response), *CRP* (regulation of gene expression, vasodilatation; aging; defense response to bacteria), *SCGB1A1* (negative regulation of IL-4, IL-5, T-cell proliferation to inflammatory response), *IL*-*17F* (metabolic; cartilage development; cytokine synthesis of IL-2, IL-6, IL-8), *TNFRSF4* (cellular defense/apoptosis; T-cell proliferation; regulation of transcriptional processes), *PLA2G2A* (lipid catabolic and small molecule metabolic function), *CREB3L3* (transcription of RNA/DNA; positive regulation of acute inflammation), *ADAMTS12* (cellular response to IL-1, TNF-α, VEGF; chondrocyte differentiation), *IL1F10* (IL-1 receptor binding; cytokine mediated signaling pathways), *NOX1* (angiogenesis; blood pressure control; metabolic function; signal transduction), *CHIA* (metabolic function; digestion; inflammatory response) and *BDKRB1* (sensory perception of pain; response to mechanical stimuli). TNFR1 as the major player of TNF-α signaling has been associated with neuropathic pain related depressive-like behavior in animal NP model along with morphological hippocampal alterations such as impaired neurogenesis and reduced neuroplasticity. It is noteworthy, that some experimental studies observed a gender-dependency of TNF signaling possibly driven by hormonal factors [19, 20]. On a peptide level, we found significantly increased IL-6, TNF-α levels along with impaired mood in our previously published study, which in part reflect the upregulated genes *IL36G*, *IL*-*17F* and *TNFRSF4*. In addition, both upregulated *IL36G* and *IL*-*17F* genes are known to modulate IL-6 synthesis [1]. Relative to the body mass index (BMI), 75% of our study population was classified as pre-obese to obese class I-III. In addition, metabolic disorder associated impairment (hypertension, diabetes, cardiac ischemia) was diagnosed in every CRPS subject of our study [1]. Immunometabolism and its potential impact on the development of chronic pain has been recognized in the past 20 years. Possible relationships to specific therapeutic approaches such as electrical neuromodulation of the brain and the spinal cord remain an under-investigated issue. Immunometabolism markers such as leptin have been associated with cellular and molecular mechanisms linking obesity to white adipose tissue (WAT) dysfunction, which in turn promotes a broad spectrum of metabolic (cardiovascular, diabetes) and/or inflammatory associated disorders (chronic pain, neuropsychiatric disorders). However, the complex puzzle of reciprocal interactions remains incomplete so far and the authors strongly recommend, to include immune-metabolic assays among other neuroinflammatory mediators in future targeted neuromodulation research for chronic pain.

These changes of the assessed transcriptome are associated with impaired sensory processing of pain, metabolic disorders and impaired mood/sleep in our CRPS cohort. Gene expression of blood cells relevant for the inflammatory host response and associated cell signal transduction were altered in our current study. However, the findings indicate an ongoing pathophysiology. If DRG stimulation interacts with CRPS pathophysiology on non-neural circulating pathways involved in the underlying mechanism of CRPS is unknown.

### Previously published animal studies addressed to genetic expression analysis at the neural interface (spinal cord and dorsal root ganglion)

Notably, only a few experimental studies exist indicating an impact of SCS/DRG patterns on genetic modification on the spinal cord/dorsal root ganglion level relevant for the neural transmission of nociception [5–8].

Alteration of the membrane function (ion flux across neuronal and glial membranes), increased peptide expression leading to hyperexcitability in DRG/spinal cord neurons (T-type Ca^2+^ currents), decreased neuronal activity at laminae I/II and opioid receptor-dependent mechanisms may contribute to the genesis of chronic neuropathic pain [1, 2, 21]. Disbalance of T-type Ca^2+^ currents (Ca_v_ 3.2 T-type channel) leads to hyperexcitability of DRG and spinal cord neurons resulting in abnormal pain/sensory perception originating from peripheral nerve injury. Although the precise mechanism remains to be fully clarified, upregulation of T-type Ca^2+^ currents may be mediated by an increase of distinct receptors subtypes (e.g. Ni^2+^ insensitive current component) leading to reduced action potential thresholds, increased action potential frequency and ectopic discharges [21].

Experimental studies have assessed possible changes in gene expression of pain-associated inflammatory peptides evoked by SCS utilizing a current-controlled stimulation protocol (50 Hz, 20 µs, 70% motor threshold, 0.3–1 mA) [2–4]. Interestingly, SCS at spine level L1 reversed experimentally induced increased RNA expression of IL-1β, IL-6, GABA B receptor 1 and sodium/potassium ATPase [5]. In another experimental setting the same working group confirmed some of the changes in gene expression in the spinal cord also occurred in the ipsilateral DRG suggesting a modulatory effect of SCS at both sites [6]. In an additional holistic gene assay approach, spinal cord genes encoding inflammatory peptides and ion channel regulatory proteins were found to respond to SCS in the presence and absence of an external noxious stimuli. Contrary, dorsal root ganglion inflammatory genes were only modified in the presence of noxious stimuli (injury) [7, 8].

Although transcriptomic assay in our study did not determine gene expression changes at the neural interface, the data presented in this study represent the first in-human DRG-SCS trial assessing changes in gene expression involved in neuroinflammatory host response in circulating blood cells [22]. Pro-inflammatory IL-1β and corresponding components (molecular/cellular) promote the development of chronic pain, metabolic disorders and neuropsychiatric disorders itself, but are frequently present in chronic pain patients, which in turn makes relational conclusion difficult due the multifunctional characteristics of cytokines/chemokines coding genes.

### Limitations

Given the multifunctional characteristics of genes assessed in our cohort, interpretation of the study findings is limited, and the application to clinical practice still requires additional work. Pro-inflammatory IL-1β and corresponding components promote the development of chronic neuropathic pain, metabolic disorders, cardiovascular diseases and neuropsychiatric disorders. On the other hand, impaired sensory perception, hypertension, heart failure, diabetes, obesity and mood disorders are highly prevalent in chronic pain patients. Even in the absence of pain-related co-morbidities, it may be useful to measure mediators associated with these co-morbidities in order to identify patients at risk to develop co-morbidities in the future. This study has several limitations including the uncontrolled design, the small-scale study cohort, lack of a sham control arm and short observation period. Furthermore, multiple variables and confounders include demographic differences (age/gender), lifestyle factors (physical activity, food intake, sleep quality), psychological issues (depression, catastrophizing) and pre-analytic variables (sample collection, storage, processing) [23]. Large-scale databank-driven phenotyping for DRG stimulation candidates is to establish a set of neuroinflammatory-based biomarkers and predictors for chronic pain amenable to this technique of neurostimulation therapy [24, 25].

## Conclusions

This is the first in-human effort to observe the feasibility of investigating the effects of DRG stimulation on the whole genome in circulating blood cells, using pathway enrichment analyses tools (Gorilla and PANTHER). Although of preliminary character, the results presented here demonstrated partial alterations in gene expression linked to inflammation and host response, in agreement with previously published observations on measured peripheral peptide levels (cytokine, oxytocin) [1]. We observed significantly altered levels of gene expression associated with inflammatory response, cell proliferation, cell signal transduction, host defense and metabolism in CRPS patients treated with L4-DRG_STIM_. The question whether DRG stimulation evoked changes in circulating transcripts are involved in the long-term effects on analgesia remains open and deserves further attention. A relational conclusion is limited due to the study protocol and may represent simply a “single shot” look at the pathophysiology in process.

Whether the differentially gene expression changes observed in our study may serve as a disease severity marker or relates to the DRG stimulation effects in CRPS subjects remains largely unknown. However, experimental studies indicate transcriptomic changes in DRG sensory neurons at lumbar spine level that have been implicated in the pathogenesis of CRPS. Chronic neuropathic pain represents a multi-network disorder of the brain, which in turn leads to a multi-functional impairment far beyond of pain. Hence, large-scale databank driven trials are recommended to re-examine the potential to serve as useful tool in clinical neurostimulation practice.

## Data Availability

The datasets used and/or analyzed during the current study are available from the corresponding author on reasonable request.

## Abbreviations

*TLR1*: toll-like receptor 1
*FFAR2*: free fatty acid receptor 2
*IL1RAP*: interleukin 1 accessory protein
*IL1RN*: interleukin 1 receptor antagonist
*C5*: complement component 5
*PKB*: PDZ binding kinase
*IL18*: interleukin 18
*CXCL2*: chemokine (C–X–C motif) ligand 2
*CCL11*: chemokine (C–C motif) ligand 11
*IL36G*: interleukin 36 gamma
*CRP*: C-reactive protein
*SCGB1A1*: secretoglobin, family 1A, member 1 (uteroglobin)
*IL*-*17F*: interleukin 17F
*TNFRSF4*: tumor necrosis factor receptor superfamily, member 4
*PLA2G2A*: phospholipase A2, group IIA (platelets, synovial fluid)
*CREB3L3*: cAMP responsive element binding protein 3-like 3
*ADAMTS12*: ADAM metallopeptidase with thrombospondin type 1 motif 12
*IL1F10*: interleukin 1 family, member 10 (theta)
*NOX1*: NADPH oxidase 1
*CHIA*: chitinase, acidic
*BDKRB1*: bradykinin receptor B1
